# Poor Fertility, Short Longevity, and Low Abundance in the Soil Seed Bank Limit Volunteer Sugarcane from Seed

**DOI:** 10.3389/fbioe.2015.00083

**Published:** 2015-06-04

**Authors:** Johann S. Pierre, Jai Perroux, Alex Whan, Anne L. Rae, Graham D. Bonnett

**Affiliations:** ^1^CSIRO Agriculture Flagship, St Lucia, QLD, Australia; ^2^Black Mountain Laboratories, CSIRO Agriculture Flagship, Canberra, ACT, Australia

**Keywords:** *Saccharum* spp., seed dormancy, seed persistence, GMO environmental risk assessment, weed competition, sugarcane, soil seed bank

## Abstract

The recent development of genetically modified sugarcane, with the aim of commercial production, requires an understanding of the potential risks of increased weediness of sugarcane as a result of spread and persistence of volunteer sugarcane. As sugarcane is propagated vegetatively from pieces of stalk and the seed plays no part in the production cycle, the fate of seed in the environment is yet to be studied. In this study, sugarcane seed samples, collected in fields over a 2-year period, were used to determine the overall level of sugarcane fertility, seed dormancy, and longevity of seed under field conditions. A survey of the soil seed bank in and around sugarcane fields was used to quantify the presence of sugarcane seeds and to identify and quantify the weeds that would compete with sugarcane seedlings. We demonstrated that under field conditions, sugarcane has low fertility and produces non-dormant seed. The viability of the seeds decayed rapidly (half-life between 1.5 and 2.1 months). This means that, in Australia, sugarcane seeds die before they encounter climatic conditions that could allow them to germinate and establish. Finally, the soil seed bank analysis revealed that there were very few sugarcane seeds relative to the large number of weed seeds that exert a large competitive effect. In conclusion, low fertility, short persistence, and poor ability to compete limit the capacity of sugarcane seed spread and persistence in the environment.

## Introduction

Sugarcane is a perennial grass cultivated in tropical and sub-tropical areas for the high sucrose content in its culm. Sugarcane is propagated vegetatively from pieces of stalk and the seed plays no part in the production cycle. In fact, the ability for sugarcane to reproduce sexually was only discovered in 1859 in Barbados [reviewed by Moore et al. (2013)], which then enabled sugarcane breeding. Modern sugarcane (*Saccharum spp*.) is a hybrid between sweet sugarcane (*S. officinarum*) and wild sugarcane (*S. spontaneum*). In recent years, new techniques have become available for sugarcane varietal improvement with the ability to introduce new genes into sugarcane to create genetically modified (GM) varieties (Lakshmanan et al., 2005).

Part of the regulatory assessment of GM sugarcane is to ensure protection of the environment. A key potential risk is increased weediness as a result of spread and persistence in its environment and how introduced traits might change this compared to conventional sugarcane. Since sugarcane is a vegetatively propagated crop, the knowledge about its sexual reproduction has been quite limited and mostly focuses on breeding improvement. Efforts have recently been made to understand sugarcane sexual reproduction in relation to its environment and the potential to increase weediness (Bonnett et al., 2007, 2008, 2010; Olivares-Villegas et al., 2010; Pierre et al., 2014).

In Australia, the extent of sugarcane flowering among a collection of parental genotypes has been evaluated for a breeding program (latitude 17°S) over 25 years and varied from 13.3 to 75.4% depending on the year (Berding et al., 2004). In commercial fields, sugarcane will flower between latitudes 17°S and 30°S in Australia but is not likely to be fertile at latitude higher than 19°S (Bonnett et al., 2010). Sugarcane fertility in breeding programs is generally low and has been evaluated for bi-parental crossing to be between 3 and 22% fertile florets (Price, 1961; Rao, 1980). When evaluated on a limited number of samples in commercial fields, fertility of sugarcane ranges from 0 to 53.3 germinated seed per gram of fuzz (a mixture of seed, its bracts, attached hairs, and parts of the rachis) (Bonnett et al., 2007). The fate of these fertile sugarcane seeds is largely unknown. Abiotic limits for seed germination regarding temperature and water requirement have been determined in a previous study (Pierre et al., 2014). It was shown that, in Australia, water availability is likely to limit seed germination at the time of their production. Consequently after release from the inflorescence, these seeds will have to retain their viability until conditions that promote their germination are met. Longevity of sugarcane seeds has been evaluated for seed conservation purposes for breeding. It has been shown that dried seeds stored in polythene bags will lose 90% of their viability when kept at ambient temperature (28°C) under lab conditions after 70 days but will keep their viability well beyond 2 years when stored at −20°C (Rao, 1980). While this study presents interesting results, which demonstrate that, under control conditions, sugarcane is a short lived seed (<1 year), it does not mimic the conditions of the environment where seed would be produced where temperature and moisture fluctuate and there is a potential for seed predation, that could drastically impact seed persistence in the soil (Blate et al., 1998; Rodríguez-Pérez and Traveset, 2007). In addition, dormancy of sugarcane seed has never been studied, even though there is no link between seed dormancy and their persistence in the soil (Thompson et al., 2003), dormancy of sugarcane seeds would prevent germination in temporarily favorable conditions that do not persist and therefore stop establishment.

In addition to physiological and environmental factors, establishment of sugarcane plants from seeds could be limited by weed competition. Among the major weeds of sugarcane are high seeding species such as *Echinochloa colona*, which could produce up to 39,000 seed per plant (Bagavathiannan et al., 2012), that can remain viable in the soil seed bank up to 12 years (Dawson and Bruns, 1975) and will germinate under a broad range of temperature and soil pH (Chauhan and Johnson, 2010). It is therefore important to characterize soil seed banks in sugarcane fields to understand how weeds growing from seed may prevent sugarcane seedling establishment.

In the work reported here, we look at some of the key factors that could either increase the likelihood of sugarcane plants establishing from seed (high viability, dormancy, and longevity) or decrease that likelihood (loss of viability, competition from other plants). We first survey the fertility of fuzz collected from areas most likely to produce viable sugarcane seeds. We then determine whether some of the most fertile samples have seeds that exhibit dormancy and using artificial soil seed banks to determine longevity under a range of conditions. Finally, we quantify and identify the range of seeds germinating from soil samples taken from sugarcane fields to understand what the likely competitive ability of sugarcane seedlings might be.

## Materials and Methods

### Studied sites

The two experimental sites were located in Queensland (Australia) at the northern limit of sugarcane cultivation. It has been demonstrated previously that sugarcane flowers and produces seed in this area (Bonnett et al., 2010). Site 1, located near Mossman (16°27′S, 145°22′E) is a sugarcane farm of 175 ha with seven cultivated varieties. Site 2, Meringa, located near Gordonvale (17°3′S, 145°46′E) is a sugarcane breeding station of 46.6 ha where about 85,000 different genotypes are planted every year (Atkin, SRA, personal communication).

### Seed material

Florets, from panicles, were collected in 2012 and 2013 between June and the end of July in the fields of sites 1 and 2. In an attempt to increase the collection of samples that may contain seeds, fields were selected based on the abundance of the flowering and the physical appearance of the panicle: wooly, not shiny, and fully expanded (i.e., mature). In 2012, only the detaching fuzz (a mixture of seed, its bracts, attached hairs, and parts of the rachis) from the panicles was collected. In 2013, the whole panicles were cut from the plant and all the fuzz was stripped from the inflorescence.

### Germination tests

Samples were weighed and germination was tested on 0.1 or 0.5 g of fuzz with three to six replicates. Fuzz was spread evenly on filter paper (Whatman, filter paper 3, 125 mm) within large Petri dishes (Corning, 150 mm × 25 mm) and watered with 20 ml of distilled water. Germination tests were conducted in an incubator (Sanyo, MLR 350HT) with constant light (198 ± 34 μmol photons m^−2^ s^−1^). In 2012, the germination experiments were conducted at 36°C and in 2013 the experiments were conducted at 30°C as this temperature was then determined to be optimal for germination (Pierre et al., 2014). The number of germinated seeds was assessed after 5 and 10 days. Germination tests were conducted under these conditions (36°C in 2012 and 30°C in 2013) in all experiments unless otherwise stated. In addition, 50 randomly chosen samples were used to estimate the average weight of florets by counting and then weighing 100 florets.

### Seed viability and dormancy

Seed viability was assessed with tetrazolium staining as described in the *Tetrazolium Testing Handbook* (2000). In each experiment, 4 batches of 20 seeds were separated from their florets and imbibed in distilled water at room temperature overnight. Seeds were bisected longitudinally through the embryo and both halves were retained for staining in a 1% tetrazolium solution for 24 h in the dark. Samples were then observed with a Leica MZ16FA stereomicroscope equipped with a DFC490 camera (Leica Microsystems Ltd., Heerbrugg). Seeds were declared viable or dead according to the staining pattern of the embryo (Association of Official Seed Analysts, 2000). To assess the level of seed dormancy, the percentage of seeds that had germinated after 10 days was compared to the percentage of viable seeds (*see statistical analyses section*). Samples were identified as having some dormant seeds when the percentage of viable seeds was statistically significantly higher than percentages of germinated seeds. Germination tests were done on 4 replicates of 20 naked seeds and 4 batches of in-floret-seeds to rule out any glume-imposed seed dormancy.

### Seed longevity under field conditions

Experiments to determine seed longevity under field conditions were conducted in 2012 and 2013. Samples used in these experiments were selected based on (i) fuzz quantity and (ii) the fuzz fertility assessed by standard germination tests. Variable quantities of fuzz were used for each sample to obtain a sufficient number of germinating seed at *T*_0_ (Table 1). Fuzz was weighed and then transferred to nylon mesh bags tied up with nylon cord before being buried in the field. For each time point/treatment, combination 5 replicates were prepared.

**Table 1:** **Seed longevity experiments samples identification, mass of fuzz buried in each bag, and number of germinated seeds at the beginning of the experiment**.

	Sample ID	Seed set	Mass (g) of fuzz per bag	Number of germinated seeds at *T*_0_
Experiment 1	2012–26	Moderate	0.6	87.2 ± 20.1
	2012–32	Low	1.2	34.6 ± 2.0
	2012–38	Moderate	0.6	87.6 ± 5.9
Experiment 2	2013–3 (high)	Low	1.5	69.6 ± 3.3
	2013–3 (low)		0.75	40.8 ± 15.8
	2013–45 (high)	High	0.22	54.2 ± 18.2
	2013–45 (low)		0.11	30 ± 22.6
	2013–49 (high)	Moderate	0.40	76 ± 3.4
	2013–49 (low)		0.20	42 ± 13.4
	2013–50 (high)	Low	0.40	43.6 ± 5.5
	2013–50 (low)		0.20	27.8 ± 13.8

Samples were buried at Mossman and Meringa sites. The Mossman site was an area used as a buffer between a creek, prone to flooding during summer (January to March), and the edge of a sugarcane field. The soil is clay-loam with an average pH of 4.6 ± 0.3. The Meringa site was located within 10 m from sugarcane fields. It has a clay-loam soil with an average pH of 5.3 ± 0.5.

In 2012 (experiment 1), 300 nylon mesh bags were buried to test the effect of time, site, and depth of burial (5–10vs. 30–40 cm) on sugarcane seed longevity of three seed samples. Samples were buried on the 19th and 20th of July 2012 and were retrieved after 1, 2, 3, 6, and 9 months. In 2013 (experiment 2), 1440 nylon mesh bags were buried to test the effect of fuzz density (high vs. low), time, site, and depth of burial (5–10 vs. 30–40 cm) on sugarcane seed longevity of four samples. Bags were buried on 25th and 26th of July 2013 and were retrieved after 1, 2, 3, 4, 6, and 10 months. The experimental plots were not sprayed with any chemicals during the course of the experiment. The plots were left mainly undisturbed except when weeds were too high and dense to locate the samples; they were then pulled out by hand or cut with a brush cutter.

Seed longevity of the samples retrieved from the field was evaluated with the standard germination tests and results were reported as average percentage of germination relative to germination at T_0_.

### Sugarcane field soil seed bank

A soil seed bank experiment was conducted from June, when sugarcane was flowering, to October 2013 to determine the presence of sugarcane seeds in the soil seed bank in and around sugarcane fields. The experiment was conducted at the same locations used for the seed longevity experiments.

At each location, three fields representative of the different stages of sugarcane breeding and production were selected to maximize the likelihood of finding sugarcane seeds in the soil seed bank. In Mossman, a fallow, a plant crop, and a ratooning crop were selected. In Meringa, a fallow and two fields where multiple varieties are grown to produce inflorescences to be used in crossing were selected. Three transects of 60 m were laid down at the edges of the selected fields and soil samples of approximately 60 cm^3^ were collected every 10 m with a drill auger (22 mm × 140 mm). The soil samples (*n* = 108) were brought back to the lab and spread evenly as a 2 cm soil layer at the top of square pots (125 mm × 150 mm) filled with University of California potting mix. Pots were then transferred to a glasshouse. Seeds were allowed to germinate and grow under non-limiting water and nutrient conditions at 28°C/20°C, which correspond to the average maximal and minimal temperature in North Queensland during winter (source: Bureau of Meteorology^1^). Seedlings and plants were identified according to their morphological characteristics.

### Statistical analyses

All the statistical analyses were conducted in the R statistical computing environment [v3.0.3; The R Foundation for Statistical Computing Core Team (2014)]. *P*-values below 0.05 were considered significant.

Significant differences in fuzz fertility between years were detected using a Wilcoxon rank sum test. Seed dormancy analyses were conducted using a one-way analysis of variance to determine whether there were any statistical differences in the percentage of germinated/viable seeds between the three groups. When *p*-value was <0.05, the Tukey HSD test was used to detect the source of differences among the groups.

For the longevity experiment, the relative percentages of germination were ln(*x* + 1) transformed before analysis based on obtaining λ ≈ 0 from the box–cox function of the MASS package (Venables and Ripley, 2002). Linear models were fitted on transformed data from the 2012 and 2013 experiments separately fitting *depth, sample, site, amount* (only in 2013) and their interaction terms. Model terms were tested for significance using the ANOVA function.

Finally, seed longevity half-life was estimated for the data from each year. The decay of seed viability was assumed to be exponential, and the decay constant was estimated by fitting a non-linear model using generalized least squares through the gnls function from the nlme package (Pinheiro et al., 2014). Half-life was then derived by dividing ln(2) by the decay constant.

## Results

### Sugarcane fertility in commercial fields

**Figure 1:**
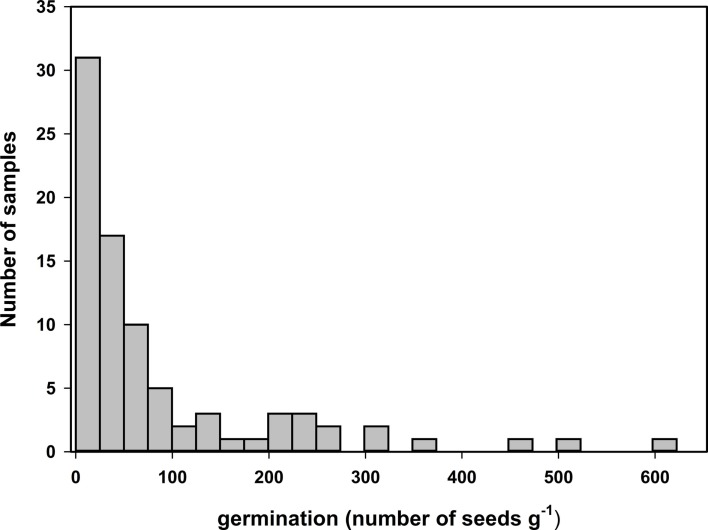
**Distribution of average number of germinated seed per gram of fuzz from 84 field samples collected in 2012 and 2013**. Germination tests were conducted at 30 or 36°C, under constant light and non-limited water conditions with either three or five technical replicates.

Germination tests of samples of seed collected over a 2-year period on sugarcane farms, and a sugarcane breeding station show the presence of viable seeds in sugarcane panicles (Figure 1). The number of seeds that germinated was highly variable, with a distribution skewed toward zero (Figure 1). The number of germinated seeds varied from 0 to 622 with an average of 88 seeds g^−1^ of fuzz. The number of germinated seeds per gram of fuzz was significantly different (*p*-value = 1.941 e^−06^) between the samples collected in 2012 and 2013, with 177 ± 30 and 43 ± 8 germinated seeds, respectively, and reflected the differences in sampling method where in 2012 only mature florets were harvested.

The weight of 100 florets was measured on 50 samples and the estimated average weight of a floret was 0.51 ± 0.07 mg. Based on this, 1 g of fuzz represents on average 1958 florets. Therefore, the average percentage of florets containing a germinating seed was 4.5 with a maximum of 31.8%.

### Sugarcane seed viability and dormancy

Viability tests carried out on 11 samples collected from the field showed that the viability of sugarcane seed varied from 65 to 97.5% with an average viability of 83.2% (Table 2). Germination tests conducted in parallel on seeds and seeds retained in the floret resulted in an average of 81.8 and 79.7% germinated seeds, respectively. Statistical analyses demonstrated that, for all the samples, the number of viable seeds was never significantly higher than the number of germinated seeds (either bare seeds or seeds retained in the floret) disproving the hypothesis that primary dormancy is present in sugarcane seeds. Sample *2012–4* had a larger number of germinated seeds than viable seeds. As this is not possible, this error may have come from either misclassifying the tetrazolium seed staining pattern for this sample, which is highly unlikely, or it was a sub-sampling artifact.

**Table 2:** **Comparison between the percentage of viable seeds with the final percentage of germinated seed and seed in the floret to assess the level of seed dormancy**.

ID	Viable seed (%)	Germinated seed (%)	Germinated seed within floret (%)	*p*-Value
2012–1	86.4 ± 2.4	85 ± 7.9	85 ± 7.9	0.983
2012–2	78.8 ± 2.4	81.3 ± 2.4	73.9 ± 4.5	0.312
2012–3	78.8 ± 3.1	86.3 ± 4.7	78.2 ± 6.8	0.492
2012–4	71.3 ± 4.3	88.8 ± 4.3	80 ± 2.0	0.026
2012–5	65 ± 3.5	73.8 ± 3.8	66.3 ± 4.7	0.302
2012–6	97.5 ± 2.5	92.5 ± 3.2	91.3 ± 3.8	0.384
2013–1	72.5 ± 3.2	76.3 ± 2.4	75 ± 3.5	0.693
2013–2	71.3 ± 4.3	58.8 ± 2.4	70 : 3.5	0.060
2013–3	92.5 ± 1.4	90 ± 2	87.5 ± 2.5	0.274
2013–4	96.3 ± 2.4	85 ± 2	87.5 ± 4.3	0.069
2013–5	86.3 ± 3.8	82.5 ± 4.3	82.5 ± 3.2	0.730

*Seeds were either germinated in the light at 30°C or 36°C under non-limiting water conditions for 10 days or imbibed and then longitudinally bisected and stained with 1% tetrazolium solution to assess their viability. Results are presented as mean ± SE (*n* = 4). *p*-Value indicates the results of one-way ANOVA between different treatments*.

### Sugarcane seed longevity under field conditions

These experiments were designed to determine how long sugarcane seed could survive in a field environment, and were assessed in two different locations over 2 years. The first experiment, conducted in 2012, was designed to assess the effect of site and the depth of burial on the longevity of different seed samples. Seed longevity was highly variable, especially during the first 3 months of seed burial. All the tested factors had a significant influence on the rate of seed decay, among them depth had the most significant effect (*p*-value <2.2 e^−16^) followed by seed sample (*p*-value = 3.8 e^−15^) and site of burial (*p*-value = 9.6 e^−05^).

**Figure 2:**
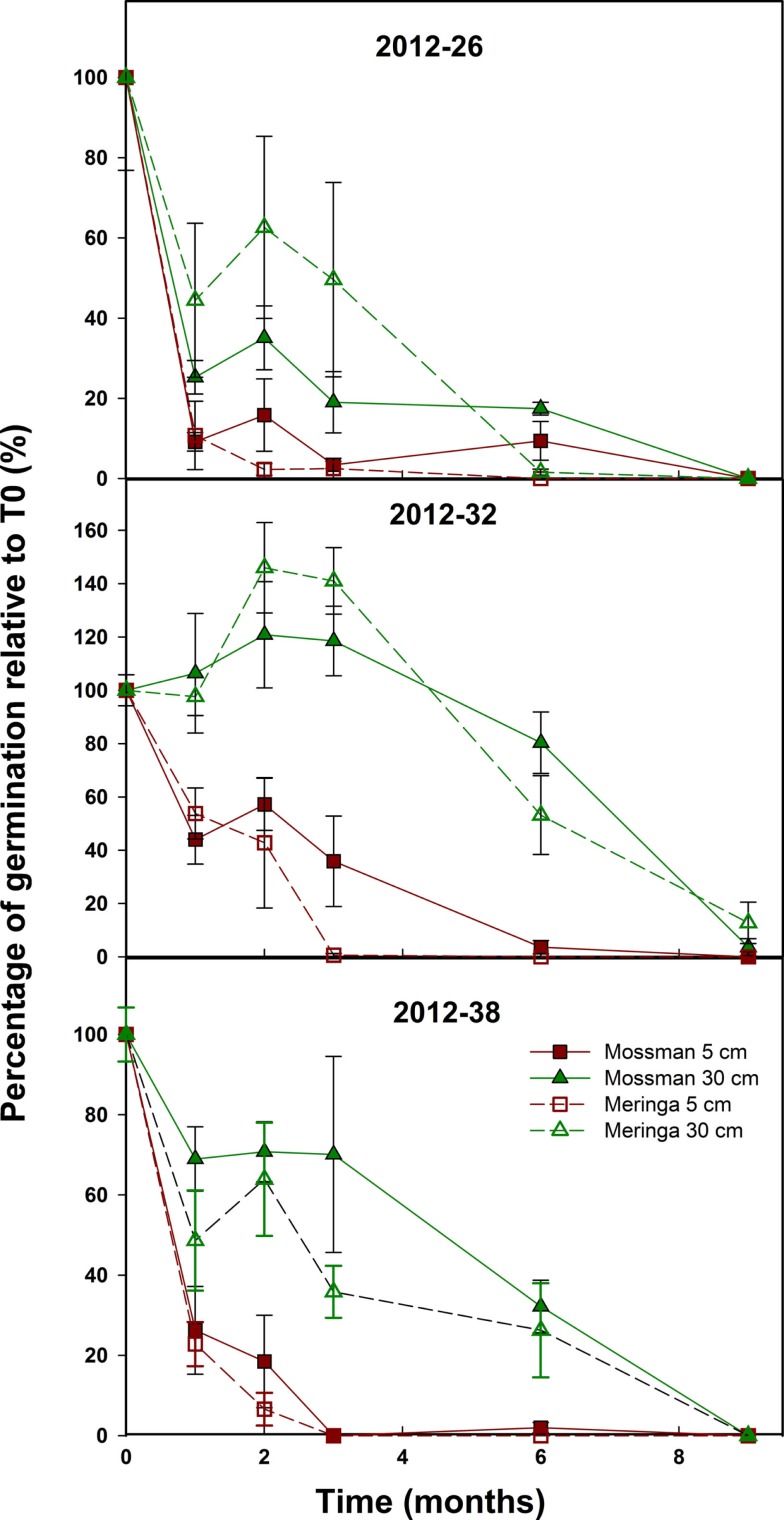
**Sugarcane seed longevity under field conditions**. The effect of site and depth of burial on sugarcane seed longevity was assessed on three field-collected samples. Symbols represent the average number of germinated seed from bags recovered from the fields each month and error bars denote SE (±). *Depth* (*p*-value <2.2 e^−16^)*, Sample* (*p*-value = 3.8 e^−15^) and *Site* (*p*-value = 9.6 e^−05^) had a significant effect on seed longevity over time.

For samples *2012–26* and *2012–38*, the general trend was a steep loss of viability from *T*_0_ with no seeds germinating after 9 months for all conditions (Figure 2). For *2012–32*, the trend was highly dependent on the depth of burial (Figure 2). The germination of the *2012–32* samples buried at 5 cm declined quickly and became null after 2–6 months while the samples buried at 30 cm germinated at the same level or even seemed to increase during the first 3 months then decreased to a very low level, close to zero after 9 months.

There was a clear effect of the depth of burial on seed longevity (Figure 2). The seeds buried at 30 cm remained viable longer than the seeds buried at 5 cm. Seed germination tended to drop dramatically after 1 month compared to the samples buried at 30 cm. At 5 cm, germination tended to be zero after 3 months while at 30 cm, this was delayed to between 6 and 9 months.

Finally, since *2012–26* and *2012–38* had a greater seed set compared to *2012–32* (Table 1), the quantity of fuzz per bag was different between samples. The correlation between seed set and longevity observed in this first experiment led us to believe that the amount of fuzz buried was a factor influencing seed longevity. Hence, in the second experiment, this factor was added in the experimental design.

**Figure 3:**
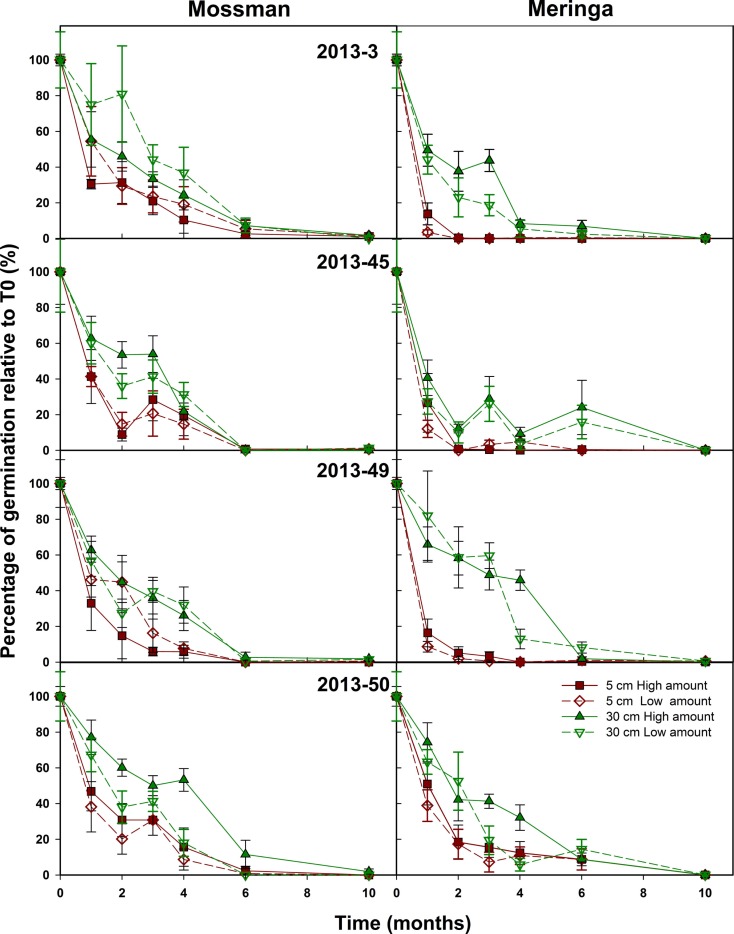
**Sugarcane seed longevity under field conditions**. The effect of amount of seed, site, and depth of burial on sugarcane seed longevity was assessed on four field-collected samples. Symbols represent the average number of germinated seed from bags recovered each month and error bars denote SE (±). *Depth* (*p*-value <2.2 e^−16^), *Site* (*p*-value <2.2 e^−16^), *Sample* (*p*-value = 2.9 e^−8^), and *Amount* (*p*-value = 0.007) had a significant effect on seed longevity over time.

In the second set of experiments, sites, depth of burial, and seed sample were factors that had a significant effect on seed longevity (Figure 3) as well as the amount of fuzz per bag that had a small but significant effect (*p*-value = 0.007) on seed longevity (Figure 3). As for experiment 1, the depth of burial had a significant impact (*p*-value <2.2 e^−16^) on the rate of seed decay that was significantly decreased when seeds were buried at 30 cm. Also, as in the 2012 experiment, seed viability was shorter at Meringa compared to Mossman (*p*-value <2.2 e^−16^) (Figure 3). The seed sample effect was significant but less important than in the first experiment (*p*-value = 2.9 e^−8^ vs. 3.8 e^−15^) (Figure 3). It appears that *2013–45*, which had the highest seed set, had the quickest rate of decay compared to *2013–50*, which had a low seed set and the slowest decay rate. *2013–3* and *2013–49*, which were considered to have a low and moderate seed set, respectively, had a life span that was not significantly different from these two extremes. In this second experiment, for most of the samples, the longevity was null or close to zero after 6 months.

**Figure 4:**
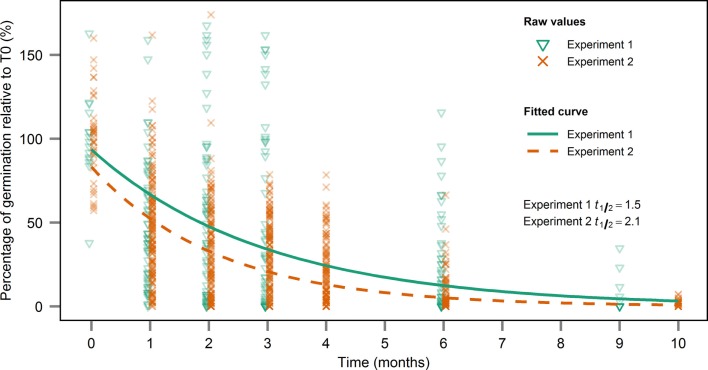
**Estimation of seed longevity half-life based on the exponential decay of seed viability**. The decay constant was estimated by fitting a non-linear model and seed longevity half-life was derived by dividing ln(2) by the decay constant.

Based on the estimated decay constant of germination over time for both years, the seed viability half-life was estimated at 1.5 months in experiment 1 and 2.1 months in experiment 2 (Figure 4).

### Sugarcane field soil seed bank

An average of 2413 ± 431 seedlings were identified every month in the soil collected in and around sugarcane fields over 5 months. The seedlings belonged to 13 different families representing a total of 29 different species (Table 3). In terms of species diversity, the Poaceae family was the most represented with 10 different species identified. In term of quantity, the Onagraceae family accounted for nearly a third of the seedlings identified with its unique representative *Ludwigia hyssopifolia* (Table 3). Finally, among the 29 species identified, 4 species (*L. hyssopifolia, O. corymbosa, Cyperus spp.*, and *E. indica*) accounted for more than 80% of the seedlings (Table 3).

**Table 3:** **Composition of soil seed banks in and around sugarcane fields over time**.

Family	Genus	Species	Months				
	
			June	July	August	September	October
Amaranthaceae	*Amaranthus*	*viridis*	0.03 (1)	_	_	_	_
Asteraceae	*Ageratum*	*conyzoides*	7.50 (230)	4.58 (145)	1.75 (20)	6.93 (133)	6.93 (168)
Asteraceae	*Eclipta*	*prostrata*	0.29 (9)	0.03 (1)	0.09 (1)	0.47 (9)	0.25 (7)
Asteraceae	*Emilia*	*sonchifolia*	1.79 (55)	0.28 (9)	1.4 (16)	0.47 (9)	0.9 (25)
Cleomaceae	*Cleome*	*aculeata*	_	1.17 (37)	_	0.21 (4)	0.18 (5)
Cyperaceae	*Cyperus*	*spp*	16.36 (502)	15.05 (476)	11.27 (129)	10.73 (206)	17.53 (486)
Fabaceae	*Mimosa*	*pudica*	0.03 (1)	0.13 (4)	0.44 (5)	0.16 (3)	0.07 (2)
Linderniaceae	*Lindernia*	*crustacea*	0.55 (17)	0.03 (1)	0.09 (1)	_	1.37 (38)
Linderniaceae	*Lindernia*	*ciliata*	0.13 (4)	_	3.32 (38)	1.09 (21)	4.87 (135)
Linderniaceae	*Lindernia*	*spp*.	1.01 (31)	1.23 (39)	_	0.94 (18)	1.48 (41)
Onagraceae	*Ludwigia*	*hyssopifolia*	28.55 (876)	28.77 (910)	25.85 (296)	28.35 (544)	28.86 (800)
Oxalidaceae	*Oxalis*	*corniculata*	_	0.03 (1)	0.17 (2)	_	0.32 (9)
Phyllanthaceae	*Phyllanthus*	*niruri*	1.30 (40)	1.55 (49)	1.40 (16)	1.88 (36)	2.06 (57)
Plantaginaceae	*Mecardonia*	*procumbens*	16.13 (495)	14.42 (456)	9.17 (105)	2.81 (54)	4.29 (119)
Poaceae	*Cynodon*	*spp*	_	0.13 (4)	_	_	_
Poaceae	*Digitaria*	*ciliaris*	1.43 (44)	0.51 (16)	4.45 (51)	2.61 (50)	1.12 (31)
Poaceae	*Dactyloctenium*	*aegyptium*	0.20 (6)	0.16 (5)	3.32 (38)	0.26 (5)	0.22 (6)
Poaceae	*Digitaria*	*sanguinalis*	_	0.63 (20)	_	_	0.94 (26)
Poaceae	*Echinochloa*	*colona*	1.24 (38)	0.57 (18)	3.93 (45)	3.91 (75)	0.43 (12)
Poaceae	*Eleusine*	*Indica*	5.44 (167)	10.43 (330)	16.68 (191)	18.19 (349)	8.12 (225)
Poaceae	*Brachiaria*	*decumbens*	0.03 (1)	0.03 (1)	_	_	_
**Poaceae**	***Saccharum***	***Sugarcane***	**0.03 (1)**	**0.22 (7)**	**_**	**_**	**_**
Poaceae	*Eleusine*	*cilianensis*	_	_	0.17 (2)	_	_
Poaceae	*Melinis*	*repens*	_	_	0.17 (2)	_	_
Rubiaceae	*Mitracarpus*	*villosus*	1.43 (44)	0.66 (21)	1.83 (21)	2.61 (50)	1.77 (49)
Rubiaceae	*Oldenlandia*	*corymbosa*	15.71 (482)	18.72 (592)	14.41 (165)	18.08 (347)	18.98 (526)
Rubiaceae	*Richardia*	*brasiliensis*	0.07 (2)	0.66 (21)	_	0.21 (4)	0.18 (5)
Rubiaceae	*Spermacoce*	*latifolia*	0.72 (22)	_	_	_	_
Solanaceae	*Solanum*	*americanum*	_	_	0.09 (1)	0.1 (2)	_
		**Total (%)**	**100 (3068)**	**100 (3163)**	**100 (1145)**	**100 (1919)**	**100 (2772)**

*Soil samples, collected in various locations and types of sugarcane field, were germinated in glasshouse at 20°C/28°C under non-limited water conditions. Results are presented as percentage of the total number of germinated/established plants identified in soil seed bank for each month. Numbers in brackets represent the absolute number of germinated/established plants of each species identified in soil seed bank*.

Sugarcane seeds represented a very small proportion of the soil seed bank in and around the sugarcane fields sampled (Table 3). In June, only one sugarcane seedling (0.03% of the soil seed bank) was found from a ratoon crop soil sample collected in Mossman (data not shown). In July, only seven sugarcane seedlings were found (0.22%) from Meringa soil samples: two were found in a sample from the fallow field and the other five were found in a sample from an arrowing paddock (data not shown). In August, September, and October samples, no sugarcane seedlings were found.

## Discussion

This paper is the first report describing the fertility of sugarcane under field conditions and the presence and persistence of sugarcane seed in artificial and natural soil seed banks.

### Sugarcane fertility and dormancy

Seeds were collected, in Queensland, around latitude 16°S–17°S, which is an area where sugarcane frequently flowers and produces seed (Bonnett et al., 2010). When tested under optimal conditions, we observed a broad spectrum of the level of germination between the collected seed samples ranging from 0 to 622 seeds per gram of fuzz. On average, 4.5% of all spikelets produced a seed that germinated. This low level of seed germination could reflect a low level of seed set or could be an underestimate of the actual number of viable seeds because some were dormant. There is no reference to sugarcane (*Saccharum spp*.) seed dormancy in the published literature. There is, however, a reference to strong primary seed dormancy in *Saccharum aegyptium*, former name of *S. spontaneum* (Poljakoff-Mayber, 1959), a wild relative of sugarcane present in an area where sugarcane is cultivated in Australia (Bonnett et al., 2008; Pierre et al., 2015). In the experiments reported here, we clearly demonstrated that dormancy is not a feature of sugarcane seed. Therefore, the low number of germinated seeds in the freshly collected samples was due to poor seed set rather than a large proportion of dormant seeds. The number of florets per sugarcane panicle has been estimated at approximately 25,000 for commercial varieties (Blackburn, 1984). Therefore, according to our data, on average, one sugarcane panicle would produce around 1125 seeds.

Comparatively, species of Poaceae used in agriculture and selected for seed or grain production have a much higher average seed set. For example, in wheat, the grain set ranges between 30 and 90% depending on the spikelet position on the plant and the variety (Ferrante et al., 2013). As an example of a GM crop species that is currently grown for commercial production, canola, with an average of 220–485 pods per plants will produce between 5000 and 8000 seeds (Cheema et al., 2001; Ozer, 2003). Canola plant density is 4–7 times higher than plant density of sugarcane (40–70 plants vs. 10 stalks/m^2^) (Garside and Bell, 2009; GRDC, 2009). Therefore, sugarcane, when it flowers, produces at least 17 times fewer seed than canola per unit area.

If seeds are released under the right conditions for their germination and establishment, then the absence of dormancy would contribute to the establishment of sugarcane seedlings. However, if seeds are released at a time of the year that does not favor seedling establishment, then it would lead to dramatic consequences for seedling survival. We demonstrated, previously, that in Australia, sugarcane seed germination at the time of their production seems to be limited by water availability (Pierre et al., 2014), therefore, for the seeds to germinate and seedlings to establish in this environment, they need to persist until periods of increased rainfall occur. Under the right conditions, establishment of a population of *Saccharum* is possible. In Panama, where wild sugarcane (*Saccharum spontaneum*) produces seeds at the time of the year of intense rainfall, recruitment of new plants from seeds contributes to its invasiveness in deforested areas (Bonnett et al., 2014). While *S. spontaneum* is present in Australian sugarcane area and some populations produce viable seeds (Bonnett et al., 2008; Pierre et al., 2015), there are no indications that the populations are expanding.

### Sugarcane longevity

When we assessed seed longevity under field conditions, we clearly showed that sugarcane seeds are short lived. Under the classification of soil seed banks by Thompson et al. (1997), sugarcane belongs to the transient category, which means that their persistence, under field conditions, does not exceed 1 year. Among all the factors we tested, depth of burial had the most striking effect on seed longevity over time in both years of experiments. It has been shown that depth of burial has a positive influence on *in situ* seed persistence (Miller and Nalewaja, 1990). Deep burial tends to protect seeds from several biotic and abiotic factors such as light (Mandoli et al., 1990) and predation (Von Euler et al., 2014), for example, but also deeper soil environments tend to have less fluctuating temperature therefore slowing down the process of seed aging (Saatkamp et al., 2011). The different depths of burial were chosen to be representative of the different stages of the sugarcane crop production. Prior to replanted fields, inversion of the soil by tillage will promote the burial of seed at 30–40 cm, while in ratooning fields, seeds will remain close to the surface. We saw that seeds buried deeper tend to remain viable longer than those buried closer to the surface but even they are not viable after 9–10 months. Since a field will only be replanted after 3–5 years, any buried seeds are not likely to be returned through subsequent tillage to the surface within the period they retain viability.

In areas where fertile seeds are produced in Australia, we have previously shown (Pierre et al., 2014) that rainfall at the time of seed maturation is likely to be a limiting factor for seed germination and seedling establishment. The onset of high rainfall in these areas is at the beginning of summer (source: Bureau of Meteorology) 6 months after seed maturation. According to our data over 2 years, when all factors were combined, the seed viability drops about 50% after 1–2 months and after 6 months viability ranges from 3 to 13%. In Coimbatore, India, sugarcane breeding uses inflorescences developed in the field; the onset of anthesis is in early November and the crossing season extends until early December (source: NHG Flowering Status data^2^ and personal communication). November is considered as a monsoonal month with 102 mm of rain (source: Climate change knowledge portal^3^). After that, the conditions are quite dry until the second monsoon, which starts in May. It takes about 30–35 days after pollination for the seed to be ripe enough to germinate (Lee and Loo, 1958); therefore, as in Australia, seed will be released at a time of the year where climatic conditions are not favorable for their germination and will need to persist in the field for about 6 months to have adequate conditions for their germination. According to our data, only 0–9.4% of the sugarcane seeds buried close to the surface (5 cm) remained viable after 6 months. Finally, in Serra do Ouro, Brazil, where climatic conditions promote flowering and production of viable seeds, crossing starts in April and extends until June (Veríssimo et al., 2002). At this time of the year, average rainfall is around 168 mm and average minimal temperature is around 24°C. In this area, sugarcane seeds could have the potential to germinate as soon as released from the mother plants, i.e., when their viability is likely to be maximal. These hypotheses, suggested by our observations of sugarcane seed longevity in Australia, highlight the need of risk assessments for each environment where future GM sugarcane may be released into.

### Natural soil seed banks

*In situ* seed longevity experiments using seed buried in a bag is a commonly used and accepted methodology to mimic the dynamics of a seed population in a soil seed bank (e.g., Egley and Chandler, 1978; Gbèhounou et al., 2003). Nevertheless, with this technique, seeds are buried in the ground at a high density, which is not necessarily representative of the situation in the field and can therefore affect seed longevity (Van Mourik et al., 2005). In our work, we tested seed longevity assuming that after dispersal, a large proportion of viable sugarcane seeds will become incorporated into the seed bank. Sugarcane seeds are small (1.5 mm × 0.64 mm, Rao, 1980) and dispersed by wind. Although there is still a debate about the influence of seed size on seed persistence, some authors point out a positive relationship between small size seeds and persistence (Hodkinson et al., 1998; Funes et al., 2007; Thompson et al., 2007). One of the reasons for this relationship is that small seeds tend to fall more easily in soil cracks and therefore reach microenvironments with more favorable conditions for their persistence. Even though we demonstrated that average sugarcane seed fertility is low, it seemed likely that with the extent of sugarcane flowering in the field, a significant proportion of seeds will be incorporated in the soil seed bank in and around sugarcane fields. The soil seed bank experiment that we conducted over a 5-month period demonstrated that viable sugarcane seeds are nearly absent in the soil seed bank. Only 8 sugarcane seedlings were found, which are a very small proportion of more than 12,000 germinated seeds identified. Sugarcane stalk density is around 10/m^2^ (Garside et al., 2005) and even assuming that only 40% of the stalks will flower (Berding et al., 2004), it represents 4450 viable seeds released/m^2^. For comparison, seed density in canola fields after harvest ranges between 2000 and 10,000 seeds/m^2^ (Lutman, 1993; Legere et al., 2001) and results in 3.9–4.9 volunteer canola plants/m^2^ after a year (Simard et al., 2002).

The gap between the estimated number of viable seeds released per plant and the number of seeds found in soil seed bank could have several explanations. One of the major differences between our artificial soil seed bank and natural soil seed bank is the density in which the seeds were packed together. For the longevity experiment, seeds were densely packed in nylon bags in a ball shape. Under these conditions, the florets containing viable seeds were surrounded by many other florets, which could have acted as a protective coat against seed degradation. In natural conditions, florets will be blown away by wind and are much less likely to form dense clumps on the ground. Therefore, the seed half-life that we obtained from our experiment probably represents a best case scenario for seed longevity, especially as the soil surface would experience more fluctuation in temperature and moisture content than 5 cm below ground.

In addition, our assessment of the soil seed bank used the seedling emergence method (Thompson and Grime, 1979). This method has proved to be efficient but appears not to produce a complete assessment of the soil seed bank flora: seeds have different germination requirements and dormant seeds are not accounted for. In our experimental setup, we selected growing conditions that will promote sugarcane seed germination and seedling establishment (Pierre et al., 2014). We ruled out seed dormancy in sugarcane, so the low number of sugarcane seedlings found in our soil seed bank experiment is not due to a bias in our experimental protocol. Nevertheless, water and nutrient were not limiting factors in our experiment, but the competition with other weeds could have prevented sugarcane seedlings from establishing. For example, *Ludwigia hyssopifolia*, the most abundant weed in our experiment, is reported to be a serious weed of rice and causes reduction in rice grain yield up to 81% in glasshouse experiments (Chauhan and Johnson, 2010). A single *L. hyssopifolia* plant can yield up to 75,000 seeds and it was demonstrated that when grown under competition, *L. hyssopifolia* has an important phenotypic plasticity and will increase its leaf-weight ratio and the stem and leaf biomass (Chauhan et al., 2011). In order to outcompete a crop when grown under high crop interference, *L. hyssopifolia* transferred 82% of its leaf biomass to the upper half of the plant compared to 25% when grown without competition. Other weeds like *Cyperus spp*., the third most represented weed in the soil seed bank experiment will decrease competition from other plants by allelopathic effect with the release of several allelochemicals compounds into the ground (Quayyum et al., 2000). Compared to these highly competitive weeds, sugarcane is a poor competitor. It has been demonstrated that, when growing from setts (stalk pieces), sugarcane is a slow growing plant (Allison et al., 2007) and would take twice this amount of time to reach canopy closure compared to other tropical crops like maize or pearl millet. Similarly, sugarcane seedlings develop very slowly compared to other closely related plants such as sorghum (unpublished observations) and so would not be adapted to compete with the weeds found in our soil seed bank. Consequently, if in this experiment potential viable sugarcane seedlings were not able to establish, it is rather unlikely that under field conditions with the same weed pressure they would do better.

## Conclusion

In conclusion, these data contribute to a baseline for competent authorities in charge of regulating GM crops, and entities developing GM sugarcane cultivars, describing the potential of transgene escape via seed. We demonstrated that the low fertility, short persistence, and poor ability to compete means, sugarcane seeds do not establish and lead to weediness in Australia. Any traits being incorporated into sugarcane that may alter the potential for weediness would be assessed against this baseline. Methods such as those used here could be employed to test any changes and to determine the baseline in other regions of the world where sugarcane is grown. The extent of hybridization if any, of *Saccharum spontaneum* with commercial sugarcane in Australia is not known. If a trait was introduced that could increase weediness potential, it may be necessary to determine if transgenes could be passed to *S. spontaneum* and what that may mean for altered seed characteristics and seedling establishment.

## Conflict of Interest Statement

The authors declare that the research was conducted in the absence of any commercial or financial relationships that could be construed as a potential conflict of interest.

## Footnotes

^1^Bureau of Meteorology (2014) Climate Data Online. http://www.bom.gov.au/climate/data/. Accessed 05 December 2014

^2^Sugarcane Breeding institute (2014). http://www.sugarcane.res.in/index.php/en/component/content/article/27-research/99-nhg-characteristics-of-parental-clones?Itemid=758. Accessed 05 December 2014.

^3^World Bank (2014) Climate Change Knowledge portal. http://sdwebx.worldbank.org/climateportal/index.cfm. Accessed 05 December 2014.

## Abbreviation

GM, genetically modified.
